# Neuroprotective effects of astaxanthin in a scopolamine-induced rat model of Alzheimer’s disease through antioxidant/anti-inflammatory pathways and opioid/benzodiazepine receptors: attenuation of Nrf2, NF-κB, and interconnected pathways

**DOI:** 10.3389/fphar.2025.1589751

**Published:** 2025-05-15

**Authors:** Zeinab Rastinpour, Sajad Fakhri, Fatemeh Abbaszadeh, Mohammad Ranjbari, Amir Kiani, Mohammed Namiq Amin, Javier Echeverría

**Affiliations:** ^1^ Student Research Committee, Kermanshah University of Medical Sciences, Kermanshah, Iran; ^2^ Pharmaceutical Sciences Research Center, Health Institute, Kermanshah University of Medical Sciences, Kermanshah, Iran; ^3^ Neurobiology Research Center, Institute of Neuroscience and Cognition, Shahid Beheshti University of Medical Sciences, Tehran, Iran; ^4^ Regenerative Medicine Research Center, Health Technology Institute, Kermanshah University of Medical Sciences, Kermanshah, Iran; ^5^ Departamento de Ciencias del Ambiente, Facultad de Química y Biología, Universidad de Santiago de Chile, Santiago, Chile

**Keywords:** astaxanthin, Alzheimer’s disease, neuroinflammation, NF-κB, matrix metalloproteinase, oxidative stress, Nrf2

## Abstract

**Background:**

Given the complexity of pathological mechanisms behind Alzheimer’s disease (AD), there is a pressing need for novel multi-targeting therapeutic agents. Astaxanthin, a natural compound with diverse biological effects, has emerged as a potential candidate in neuronal diseases.

**Purpose:**

This study aimed to evaluate the neuroprotective effects of astaxanthin in a scopolamine-induced rat model of AD.

**Materials and methods:**

In total, 36 male Wistar rats were divided into six groups, including a control group receiving normal saline, a negative control group treated with scopolamine (1 mg/kg), and two groups receiving astaxanthin at doses of 5 and 10 mg/kg. Additionally, two groups were pre-treated with naloxone (0.1 mg/kg) or flumazenil (0.5 mg/kg) to block opioid and benzodiazepine receptors, respectively, followed by receiving the most effective dose of astaxanthin (i.e., 10 mg/kg). Treatments were administered via intraperitoneal injection for 14 consecutive days and behavioral tests were done. Biochemical analyses, zymography, Western blotting, and histopathological examinations were also performed.

**Results and discussion:**

Astaxanthin treatment significantly improved cognitive function, enhanced plasma antioxidant capacity by increasing catalase and glutathione levels, and reduced nitrite levels. It also increased serum activity of matrix metalloproteinase 2 (MMP-2), while decreasing MMP-9, increasing the expression of nuclear factor erythroid 2–related factor 2 (Nrf-2) and decreasing nuclear factor kappa B (NF-κB) in hippocampal tissue. Histopathological findings indicated reduced hippocampal damage after astaxanthin administration. The aforementioned protective effects of astaxanthin were reversed by naloxone and flumazenil.

**Conclusion:**

Astaxanthin demonstrates protective effects against scopolamine-induced AD through its neuroprotective, antioxidant, and anti-inflammatory properties, potentially involving interactions with opioid and benzodiazepine receptors.

## 1 Introduction

Alzheimer’s disease (AD) is the leading cause of dementia worldwide and presents a significant public health challenge due to its complex biology and the lack of effective treatments. Progressive cognitive decline, memory deficits, and behavioral changes primarily characterize the disease. AD is multifactorial, involving various pathways that lead to synaptic dysfunction and neuronal death (Scheltens et al., 2021). Key contributors to this pathology include the accumulation of amyloid-β (Aβ) plaques, hyperphosphorylation of tau protein, neuroinflammation (Smirnov and Galasko, 2022), and oxidative stress (Bai et al., 2022). These processes disrupt critical neuronal networks, particularly in the cerebral cortex and hippocampus, which are vital for memory and learning (Eichenbaum, 2004). Oxidative stress is an important factor in the pathophysiology of AD. The brain experiences an imbalance between pro-oxidants and antioxidants due to the excessive synthesis of reactive oxygen species (ROS) and reactive nitrogen species (RNS), which is often caused by Aβ aggregation (Zhao and Zhao, 2013). This imbalance contributes to the gradual neurodegeneration seen in AD by aggravating neuronal damage, promoting lipid peroxidation, and accelerating the creation of neurofibrillary tangles. The antioxidant defense mechanisms act through combating ROS and enzymes of stages I and II of biotransformation. Considerable increase in catalase activity further reveals the effects of drugs in combating oxidative stress (Ishak et al., 2024). Age is a significant risk factor for AD, and the reduced ability of the body to produce antioxidants makes neurons even more susceptible to oxidative damage (Moneim, 2015). Furthermore, research has shown that despite elevated levels of oxidative stress markers in the brains of individuals with AD, the nuclear translocation of nuclear factor erythroid 2-related factor 2 (Nrf2) is often impaired. This impairment contributes to decreased expression of antioxidant genes, further worsening oxidative damage (Khan et al., 2020; Davies et al., 2021).

Neuroinflammation is another critical factor in AD pathology. It involves the activation of nuclear factor kappa B (NF-κB) and the release of pro-inflammatory cytokines, which can exacerbate oxidative stress and neuronal injury (Sun et al., 2022; Thakur et al., 2023). Neuroinflammatory responses can disrupt the blood-brain barrier (BBB), allowing peripheral immune cells to infiltrate the central nervous system (CNS), thereby intensifying neuroinflammation. MMPs are involved in this process; they can degrade extracellular matrix components and facilitate inflammatory cell migration into the brain (Duits et al., 2015; Behl et al., 2021; Zarneshan et al., 2025).

On the other hand, opioid receptors (µ, δ, and κ) play a significant role in cognitive functions, including learning and memory. In individuals with AD, dysregulation of these receptors has been noted, especially in brain regions vital for cognition, such as the hippocampus and cortex (Klenowski et al., 2015; Tanguturi and Streicher, 2023). Studies have shown that signaling pathways that pass through opioid receptors can affect the expression of β-secretase and γ-secretase enzymes, which are essential for the production of Aβ peptides, as a key factor in the development of AD (Teng et al., 2010; Pellissier et al., 2018). This indicates that opioid receptors may influence amyloidogenic pathways and contribute to the neurodegenerative processes associated with AD. Besides, gamma-aminobutyric acid type A (GABA-A) receptors are integral to maintaining the balance between excitatory and inhibitory signals in the brain. These receptors are also involved in synaptic plasticity, which is essential for learning and memory (Sakimoto et al., 2021). Given these insights, enhancing brain antioxidant mechanisms and modulating neuroinflammatory processes, as well as opioid/benzodiazepine receptors appears to be a suitable therapeutic approach for AD. It urges the need for finding novel multi-targeting agents in combating AD.

Astaxanthin, a naturally occurring carotenoid pigment abundant in algae and shellfish, has garnered significant interest due to its potent antioxidant properties (Fakhri et al., 2021b). What distinguishes astaxanthin from many other antioxidants is its unique chemical structure, which allows it to effectively cross the blood-brain barrier and provide direct protection to the CNS (Galasso et al., 2018; Fakhri et al., 2021a; Medoro et al., 2023). Preclinical studies have demonstrated that astaxanthin can reduce oxidative stress, inhibit inflammatory responses, and prevent neuronal apoptosis in models of neurodegenerative diseases (Fakhri et al., 2019a; Fakhri et al., 2019b). Its ability to neutralize ROS and enhance mitochondrial function makes it a promising candidate for countering oxidative damage associated with AD (Taksima et al., 2019). Moreover, research in animal models of AD has shown that astaxanthin’s neuroprotective and anti-inflammatory effects are correlated with improved cognitive performance, further highlighting its potential therapeutic benefits (Rahman et al., 2019; El Sayed and Ghoneum, 2020).

While the existing evidence supports the neuroprotective potential of astaxanthin, a detailed understanding of its major effects on oxidative stress and neuroinflammation, along with interactions with the GABA-A and opioid receptor systems, is crucial for developing effective treatment strategies for AD. The objective of this study was to explore the antioxidant and anti-inflammatory effects of astaxanthin in a rat model of AD induced by scopolamine, with a particular emphasis on its interactions with GABA-A and opioid receptors.

## 2 Materials and methods

### 2.1 Chemicals

Astaxanthin was purchased from Sigma-Aldrich Company (St. Louis, MO), and naloxone (Nal), an opioid antagonist, was sourced from Caspian Tamin Pharmaceutical Company (Gilan, Iran). Flumazenil (Flu), a benzodiazepine receptor antagonist obtained from Hameln (Hameln, Germany). Scopolamine, an anticholinergic agent, and astaxanthin were procured from Merck Company (Darmstadt, Germany).

### 2.2 Animals

Four-week-old male Wistar rats weighing 200–250 g were used in this research. The animals were kept in a controlled environment with a 12-h light/dark cycle, a steady temperature of 22°C ± 2°C, and a relative humidity of 65% ± 2%. To guarantee a constant intake of nutrients, the rats were given free access to water and a regular laboratory chow diet. In total, 36 rats were used in the investigation; they were divided into six experimental groups at random (*n* = 6/group). Under the ethical approval number IR.KUMS.REC.1400.546, the Institutional Animal Care and Use Committee (IACUC) of Kermanshah University of Medical Sciences authorized all animal handling, care, and experimental methods.

### 2.3 Experiment design

Male Wistar rats were divided into six groups and treated for 14 days. The control group (received only sodium chloride, i.p.), Scop (negative control group, received scopolamine 1 mg/kg/day, i.p.), two groups of AST 5 (receiving scopolamine 1 mg/kg/day + astaxanthin 5 mg/kg/day, i.p.) and AST 10 (receiving scopolamine 1 mg/kg/day + astaxanthin 10 mg/kg/day, i.p.). Groups V, Nal (receiving scopolamine 1 mg/kg/day + naloxone 0.1 mg/kg/day + astaxanthin 10 mg/kg/day, i.p.) and group VI Flu (receiving scopolamine 1 mg/kg/day + flumazenil 0.5 mg/kg/day + astaxanthin 10 mg/kg/day, i.p.). Astaxanthin was administered 30 min before scopolamine, while Flu and Nal were injected 15 min before the administration of astaxanthin. This treatment protocol was maintained for 14 days.

### 2.4 Behavioral tests

Behavioral evaluations were carried out on days 6, 7, 13, and 14.

#### 2.4.1 Open field test

The open field test is a valuable behavioral assessment for evaluating locomotor activity and anxiety-like behaviors in rats. The open field box was divided into equal squares. During the test, each rat was placed in the center square of the apparatus. Several key behavioral metrics, including the total number of crossings, as an indicator of exploratory behavior, the frequency of rearing (standing on their hind legs, which reflects curiosity and exploration), and grooming (cleaning itself, which can indicate stress levels or comfort) are recorded for 5 min (Prut and Belzung, 2003). After each trial test, the apparatus was thoroughly cleaned with 70% ethanol to eliminate residual odors that could influence the rats’ behavior. The study incorporated acclimation sessions on days 6 and 13 of the treatment protocol. These sessions were designed to familiarize the rats with the open field box and reduce potential anxiety or novelty effects during the evaluation sessions that occur on days 7 and 14 (Gould et al., 2009).

#### 2.4.2 Passive avoidance test

One well-known technique for assessing memory and learning in mouse models of neurological diseases is the passive avoidance test (Ghafarimoghadam et al., 2022). The experimental apparatus consists of two equal-sized compartments: a light compartment and a dark compartment, separated by a gate. Both compartments have stainless steel floors that are electrically wired. On days 6 and 13, rats were placed in front of the open gate leading to the dark compartment. Upon entering the dark compartment, an electric shock (1.5 mA for 3 s) is administered. The time taken for the rat to move from the light compartment to the dark compartment is recorded as the initial transfer latency (ITL). On Days 7 and 14, the test was repeated to assess memory retention. The time taken for the rat to enter the dark compartment on these days was referred to as the step-through latency (STL). A longer delay in entering the dark compartment indicates better memory recall of the unpleasant experience associated with the electric shock (Zarneshan et al., 2025).

#### 2.4.3 Elevated plus maze

The elevated plus maze is a commonly used experimental device for evaluating anxiety-related behaviors and cognitive abilities in animal models. Its structure consists of a cross-shaped platform elevated 50 cm above the ground, featuring two enclosed arms (50 cm × 10 cm × 40 cm) and two orthogonal open arms (10 cm × 40 cm), connected by a central platform (10 cm × 10 cm). During the learning session on day 6, rats were placed in an unprotected open arm, and the duration until they entered an enclosed arm was measured as the ITL. The experiment was conducted again on days 7 and 14 to assess memory retrieval, and the duration taken to transition from the open arm to the enclosed arm is documented as transfer latency (TL). A longer TL suggests improved memory recall and reduced anxiety (Danduga and Kola, 2024).

### 2.5 Biochemical analysis

#### 2.5.1 Measurement of catalase level

The catalase activity was evaluated by the Aebi method, a recognized approach for quantifying the enzyme’s action. For the experiment, 20 µL of serum was incorporated into a solution of 65 mM hydrogen peroxide. The reaction mixture was incubated at 25°C for 4 min. Subsequently, 100 µL of 4.32 mM ammonium molybdate was added to halt the process. The absorbance of the resultant solution was quantified at 405 nm with an ELISA reader. The percentage difference in optical absorbance between the experimental groups and the control group was determined to evaluate the levels of catalase (Aebi, 1984).

#### 2.5.2 Measurement of glutathione level

The glutathione level was measured using Ellman’s technique, which involves the interaction of thiol groups with 5,5′-dithiobis-(2-nitrobenzoic acid) (DTNB) to produce a yellow-colored product quantifiable by spectrophotometry (Ellman, 1959). For the experiment, 50 µL of phosphate buffer (pH 7.4) and 60 µL of the sample were introduced into each well. Subsequently, 100 µL of DTNB reagent was added, and the mixture was incubated at 37°C for 10 min to facilitate the reaction. The absorbance of the reaction product was then quantified at 412 nm using an ELISA reader. The differences in OD expressed as a percentage between the normal control and other groups (sample) were calculated using the following formula (Fakhri et al., 2022b).

$$\text{Concentration difference }\left(\%\right)=\left(\left(C_{\text{control}}\;–\;C_{\text{sample}}\right)\;/\;C_{\text{sample}}\right)\times 100$$

#### 2.5.3 Measurement of nitrite

The nitrite levels were assessed by the Griess reagent technique, a commonly used colorimetric test for identifying nitrite concentrations. Nitrate was first reduced to nitrite, which then reacted with the Griess reagent to produce a chromophoric azo molecule. The procedure commenced with deproteinization using zinc sulfate, followed by centrifugation at 10,000 rpm for 10 min. Next, 100 µL of vanadium chloride solution was added to the serum supernatant to convert nitrates into nitrites. The resulting mixture was then combined with Griess reagent (sulfanilamide and ethylene diamine dihydrochloride) and incubated for 30 min at 37°C. Finally, the color intensity was measured at a wavelength of 540 nm (Berkels et al., 2004).

### 2.6 Zymography

To assess gelatinase activity, serum samples containing 100 μg of total protein were loaded onto SDS-PAGE gels supplemented with 0.1% gelatin. The gel was electrophoresed at 150 V. Following this, the gel was washed with a buffer solution containing 2.5% Triton X-100 in 50 mM Tris-HCl for 1 h. The washed gel was then incubated at 37°C for 18 h in a buffer containing 10 mM calcium chloride (CaCl_2_), 0.02% NaN_3_, and 0.15 M sodium chloride (NaCl) in 50 mM HCl. After incubation, the gel was stained with Coomassie blue, which binds to proteins and allows the visualization of protein bands. To enhance the contrast between the stained protein bands and the clear areas, excess dye was removed from the gel using a solution of 5% acetic acid and 7% methanol. Finally, the bands were quantified using ImageJ software (Hashemi et al., 2024).

### 2.7 Western blot analysis

For Western blotting, hippocampal tissues were lysed in RIPA buffer and centrifuged at 14,000 rpm for 20 min at 4°C. According to the manufacturer’s instructions, protein concentrations were determined using the Bradford Protein Quantification kit (DB0017, DNAbioTech, Iran). A total of 40 μg of lysate was loaded onto SDS-PAGE. The proteins were transferred to a PVDF membrane (Bio-Rad Laboratories, CA, United States) and blocked with 5% BSA (Sigma Aldrich, MO, United States) in 0.1% Tween 20 for 1 h. Membranes were then incubated overnight at 4°C with anti- NF-κB (1:1,000, Abcam), anti-Nrf2 (1:1,000, Biorbyt), and anti-β-actin antibodies (1:2500, Abcam). After washing with TBST, the membranes were incubated for 1 h with goat anti-rabbit IgG H&L (HRP) secondary antibody (1:10,000, Abcam). The protein bands were visualized using a chemiluminescence kit and X-ray films. Densitometric analysis was then conducted using ImageJ software (NIH, United States) (Fakhri et al., 2019b).

### 2.8 Histology

On the 14th day, whole brains were fixed in 10% formalin and subsequently dehydrated using ethanol at varying concentrations before being embedded in paraffin. Thin sections (5 µm) of the hippocampus were stained with hematoxylin and eosin (H&E). An Olympus CX23 light microscope, a Dino-Lite camera, and DinoCapture 2.0 software were used for imaging. The histopathological evaluation was performed by a blinded experimenter (El-Missiry et al., 2018).

### 2.9 Statistical analysis

Statistical analyses were performed using GraphPad Prism software (version 8.4.3). To assess the normality distribution of the data, the Shapiro test was conducted. One-way or two-way analysis of variance (ANOVA), followed by Tukey or Bonferroni *post hoc* tests, was employed. A *P*-value of less than 0.05 (*P* < 0.05) was considered statistically significant, and results are presented as mean ± standard error of the mean (SEM).

## 3 Results

### 3.1 Open-field test

**FIGURE 1:**
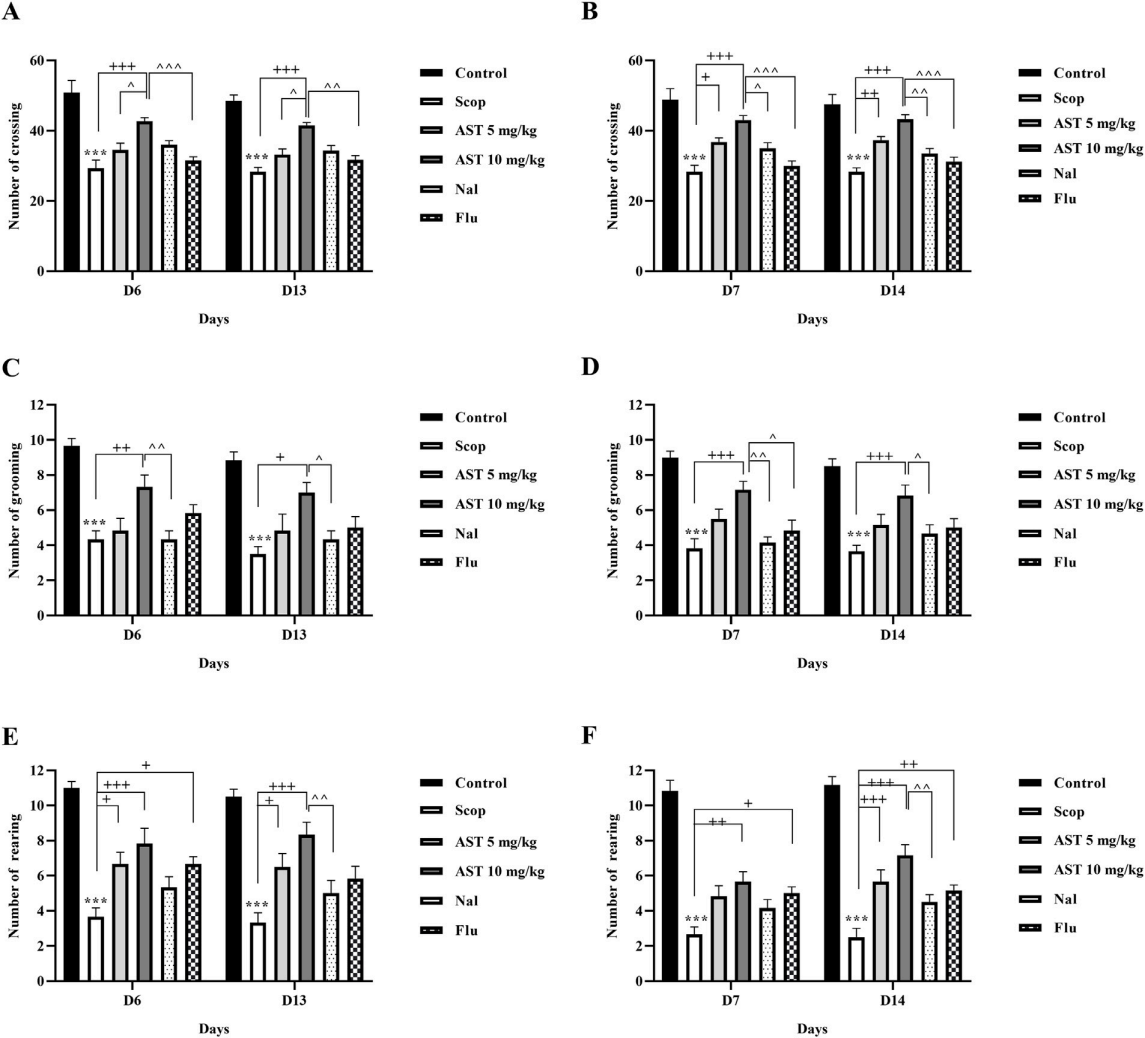
Effect of astaxanthin and co-administration with Flu and Nal on open field test in a rat model of AD. Day 6 and day 13: **(A)** number of crossings, **(C)** number of grooming, and **(E)** number of rearing. Day 7 and day 14: **(B)** number of crossings, **(D)** number of grooming, and **(F)** number of rearing. In Figure Flu: Flumazenil + AST 10 mg/kg; and Nal: Naloxone + AST 10 mg/kg; AST: Astaxanthin; Scop: Scopolamine. ^***^ *P* < 0.001 compared with control group, ^+^ *P* < 0.05, ^++^ *P* < 0.01, ^+++^ *P* < 0.001 compared with Scop group. ^*P* < 0.05, ^^*P* < 0.01, ^^^*P* < 0.001 compared with astaxanthin. Data are reported as mean ± SEM (*n* = 6).

The exploratory behavior of rats was assessed by the open-field test, focusing on the frequency of crossings (Figures 1A,B), grooming (Figures 1C,D), and rearing (Figures 1E,F) activities. Administration of scopolamine, a drug known to induce cognitive and motor impairments, led to significant reductions in all measured behaviors throughout the experiment in the Scop group (*P* < 0.001). Conversely, administration of astaxanthin, particularly at 10 mg/kg, significantly improved impairments induced by scopolamine (*P* < 0.05). Pre-treatment with Flu (GABA-A receptor antagonist) and Na1 (opioid receptor antagonist), significantly reduced the therapeutic effects of astaxanthin on the test days (*P* < 0.05).

### 3.2 Passive avoidance test

**FIGURE 2:**
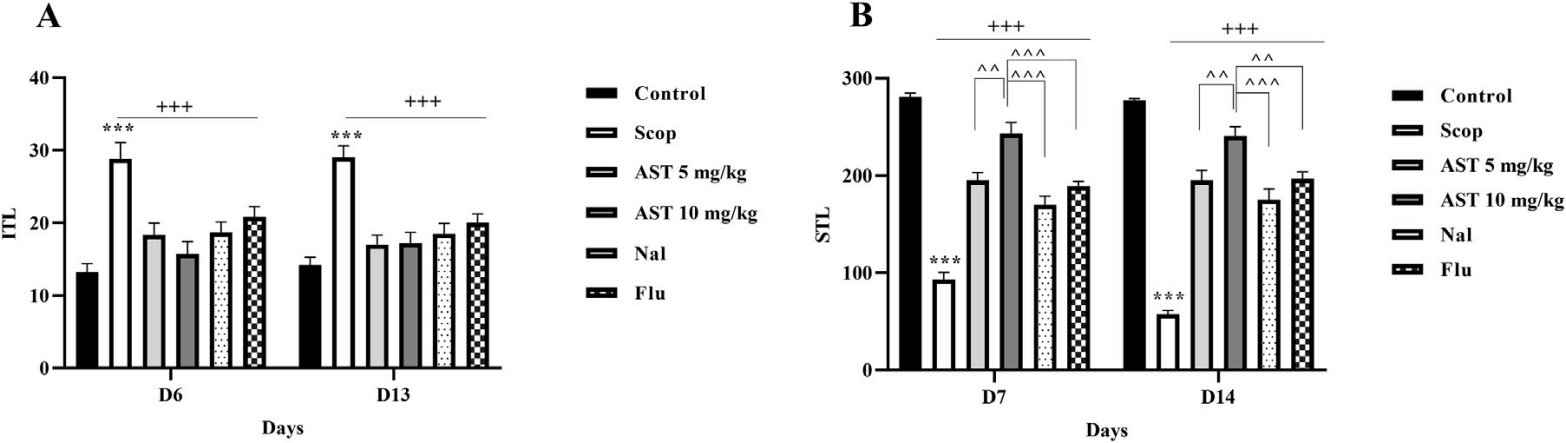
Effect of astaxanthin and co-administration with Flu and Nal on passive avoidance learning and memory in a rat model of AD. **(A)** Initial transfer latency (ITL-Day 6 and day 13) and **(B)** step-through latency (STL-Day 7 and day 14). In Figure Flu: Flumazenil + AST 10 mg/kg; and Nal: Naloxone + AST 10 mg/kg; AST: Astaxanthin; Scop: Scopolamine. ^***^ *P* < 0.001 compared with control group, ^+++^ *P* < 0.001, compared with Scop group, ^^*P* < 0.01, ^^^*P* < 0.001 compared with astaxanthin. Data are reported as mean ± SEM (*n* = 6).

The results from the shuttle test indicated that the negative control group (Scop group) showed a significant increase in ITL (*P* < 0.001, Figure 2A). Treatment with both doses of astaxanthin (5 and 10 mg/kg) resulted in a significant reduction in ITL on days 6 and 13 (*P* < 0.001). Furthermore, scopolamine treatment significantly decreased STL (*P* < 0.001). In contrast, astaxanthin treatment resulted in a significant increase in STL compared to the scopolamine group on days 7 and 14 (*P* < 0.001). Additionally, a significant reversal of the effects of astaxanthin (10 mg/kg) was observed in groups pre-treated with Flu and Nal (*P* < 0.01).

### 3.3 Elevated plus maze

**FIGURE 3:**
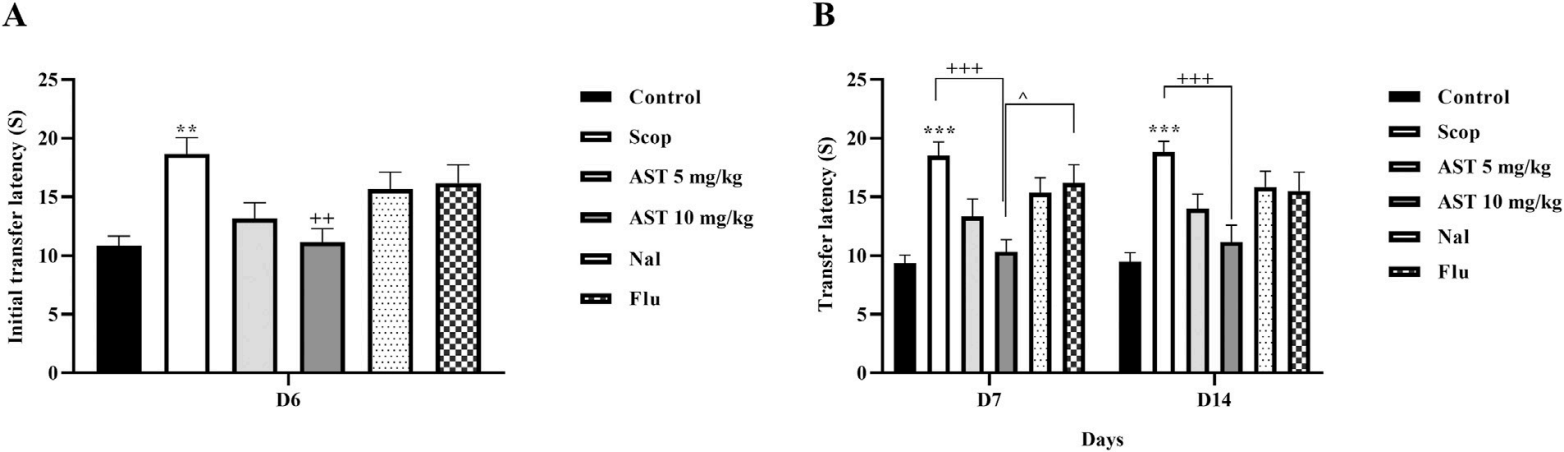
Effect of astaxanthin and co-administration with Flu and Nal on elevated plus maze and memory in a rat model of AD. **(A)** initial transfer latency (Day 6) and **(B)** transfer latency (Day 7 and day 14). In Figure Flu: Flumazenil + AST 10 mg/kg; and Nal: Naloxone + AST 10 mg/kg; AST: Astaxanthin; Scop: Scopolamine. ^**^ *P* < 0.01, ^***^ *P* < 0.001 compared with control group, ^++^ *P* < 0.01, ^+++^ *P* < 0.001 compared with Scop group, ^^^ *P* < 0.05 compared with astaxanthin. Data are reported as mean ± SEM (*n* = 6).

The results indicated that scopolamine treatment resulted in a notable increase in ITL in the scopolamine group compared to the control group (*P* < 0.01, Figure 3A), suggesting an increased anxiety or poor memory retention. In contrast, the administration of astaxanthin, especially at a dosage of 10 mg/kg, led to a significant reduction in ITL (*P* < 0.01), demonstrating its cognitive-enhancing properties. Notably, pre-treatment with the antagonists Flu and Nal reversed these positive effects. Furthermore, rats treated with scopolamine exhibited significant increases in TL on days 7 and 14 (*P* < 0.001, Figure 3B), indicating poor memory retention. In contrast, the astaxanthin-treated group showed significant decreases in TL, effectively counteracting the memory impairments caused by scopolamine (*P* < 0.001). Pre-treatment with the antagonists Flu and Nal diminished the positive effects of astaxanthin on TL, and the effects of Flu were statistically significant (*P* < 0.05).

### 3.4 Determination of antioxidant markers

**FIGURE 4:**
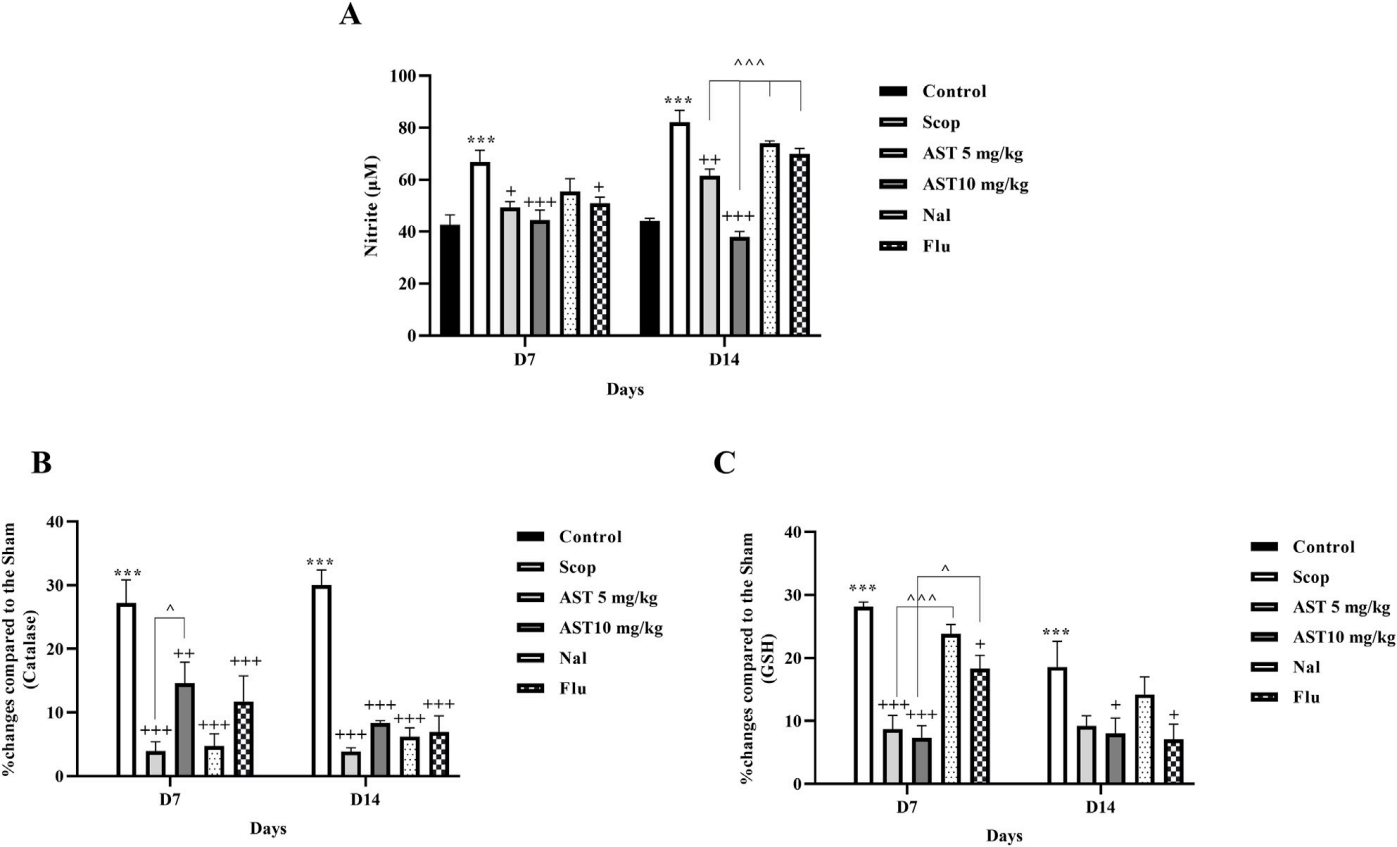
Effect of astaxanthin and co-administration with Flu and Nal on oxidative stress in a rat model of AD. **(A)** Nitrite, **(B)** catalase, and **(C)** GSH. In Figure Flu: Flumazenil + AST 10 mg/kg; and Nal: Naloxone + AST 10 mg/kg; AST: Astaxanthin; Scop: Scopolamine. ^***^ *P* < 0.001 compared with control group, ^+^ *P* < 0.05, ^++^ *P* < 0.01, ^+++^ *P* < 0.001 compared with Scop group, and ^*P* < 0.05, ^^^*P* < 0.001 compared with astaxanthin. Data are reported as mean ± SEM.

Compared to the control group, the Scop group demonstrated a significant increase in serum nitrite levels (*P* < 0.001, Figure 4A), suggesting enhanced nitrite production. Furthermore, the Scop group displayed markedly lower levels of serum catalase (*P* < 0.001, Figure 4B) and glutathione (*P* < 0.001, Figure 4C). In contrast, treatment with astaxanthin, particularly at a concentration of 10 mg/kg, led to significant restoration of these biochemical markers. However, pre-treatment with the antagonists Flu and Nal reduced the beneficial effects of astaxanthin (*P* < 0.05).

### 3.5 Zymography

**FIGURE 5:**
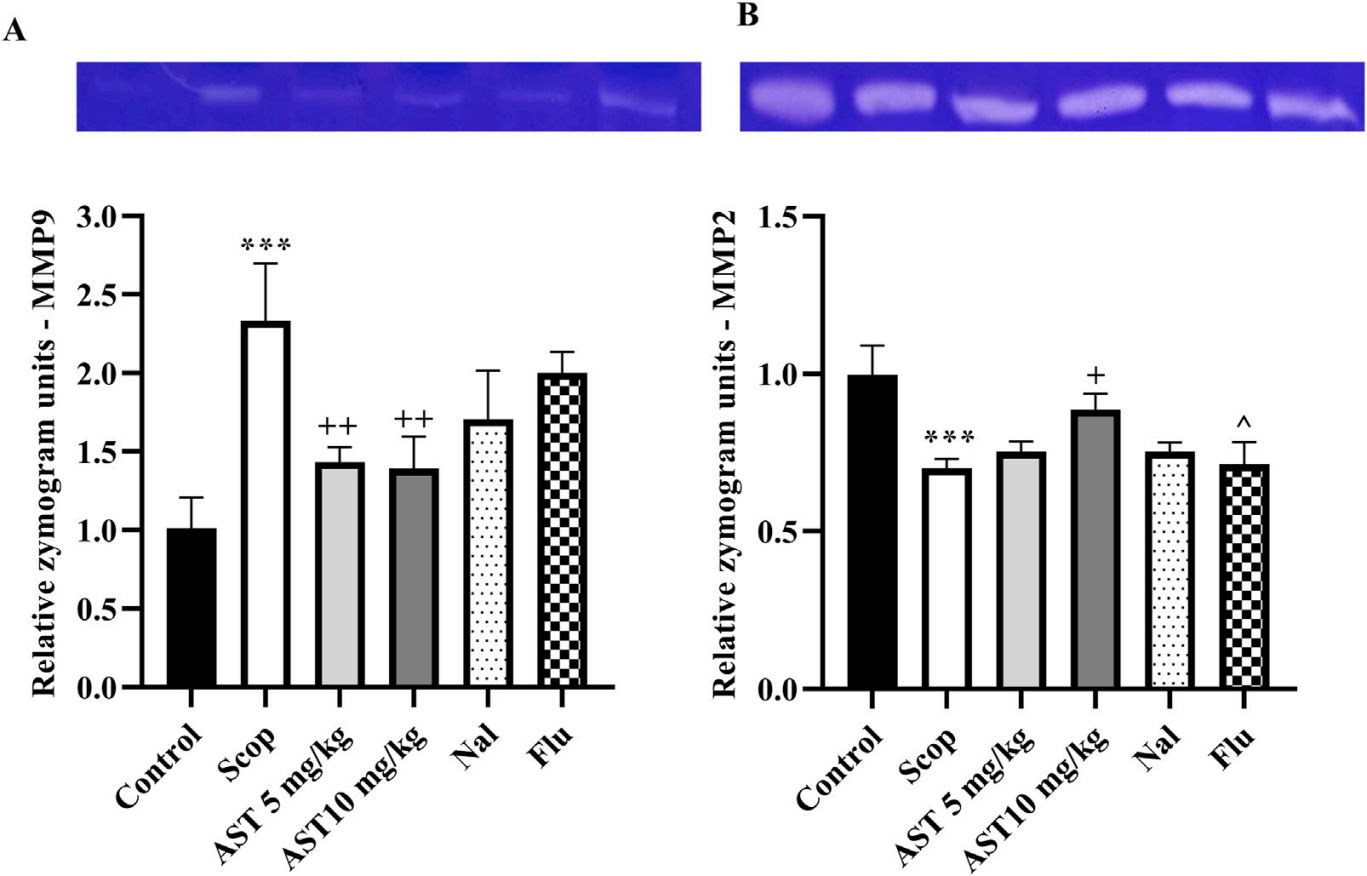
Effect of astaxanthin and co-administration with Flu and Nal on MMP in a rat model of AD. **(A)** MMP-9, and **(B)** MMP-2. In Figure Flu: Flumazenil + AST 10 mg/kg; and Nal: Naloxone + AST 10 mg/kg; AST: Astaxanthin; Scop: Scopolamine. ^***^ *P* < 0.001 compared with control group, ^+^ *P* < 0.05, ^++^ *P* < 0.01, compared with Scop group, and ^^^ *P* < 0.05 compared with astaxanthin. Data are reported as mean ± SEM.

Compared to the control group, the Scop group demonstrated a significant increase in MMP-9 (*P* < 0.001, Figure 5A) and a reduction in MMP-2 levels (*P* < 0.001, Figure 5B). In contrast, treatment with astaxanthin, particularly at a concentration of 10 mg/kg, led to significant restoration of these levels (*P* < 0.05). However, pre-treatment with the antagonists Flu and Nal reduced the beneficial effects of astaxanthin (*P* < 0.05).

### 3.6 Histology

**FIGURE 6:**
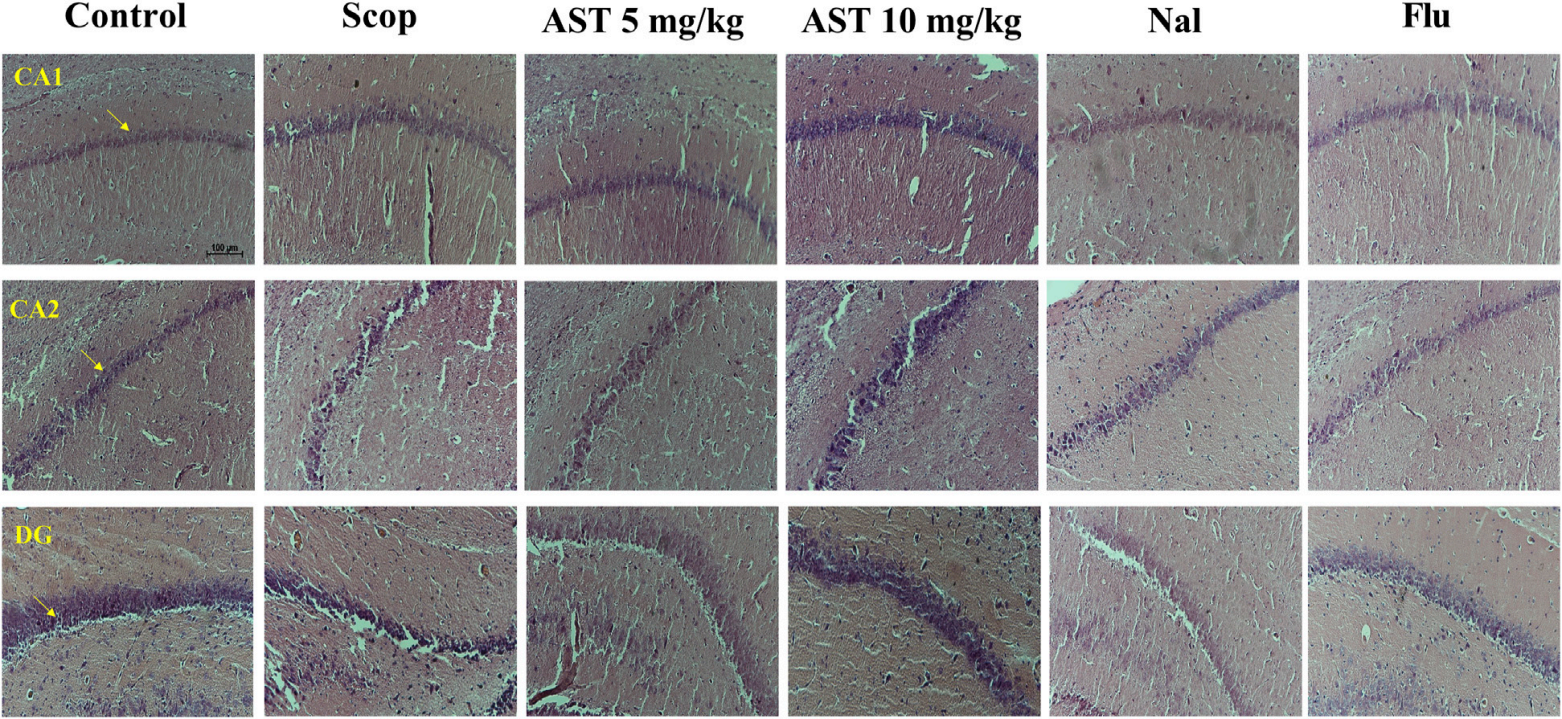
Effect of astaxanthin and co-administration with Flu and Nal on hippocampus tissue (CA1, CA2, and DG) changes in a rat model of AD, Arrows indicate healthy neurons (X20). In Figure Flu: Flumazenil + AST 10 mg/kg; and Nal: Naloxone + AST 10 mg/kg; AST: Astaxanthin; Scop: Scopolamine.

Administration of Scop led to significant neuronal loss in the hippocampal region (CA1, CA2, and DG). However, treatment with astaxanthin demonstrated a protective effect on these neurons, helping to preserve their function and integrity. On the other hand, the administration of Flu and Nal appeared to reverse the protective effects of astaxanthin (Figure 6).

### 3.7 Western blot

**FIGURE 7:**
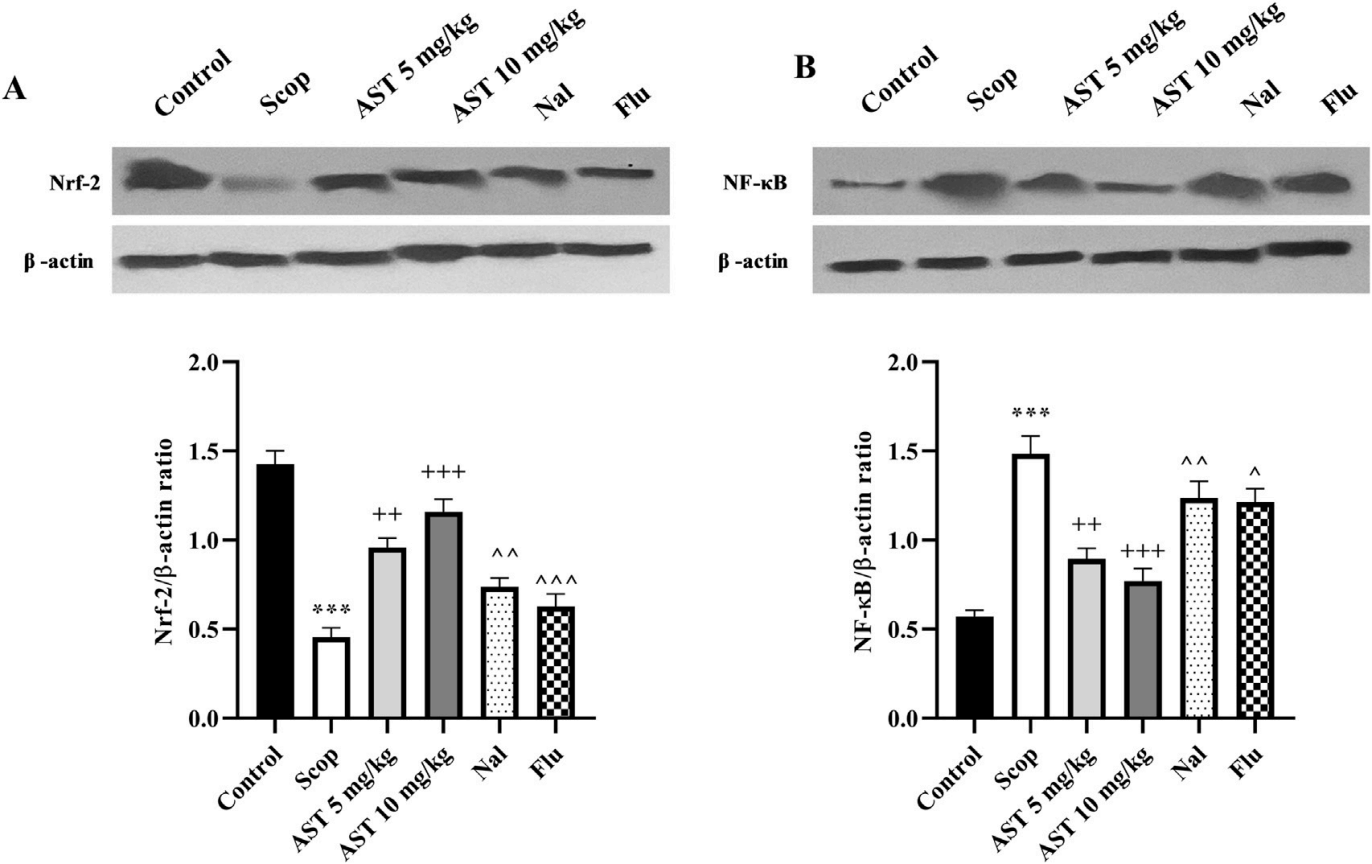
Effect of astaxanthin and co-administration with Flu and Nal on the expression levels of Nrf-2 and NF-κB in a rat model of AD. **(A)** Nrf-2, and **(B)** NF-κB. In Figure Flu: Flumazenil + AST 10 mg/kg; and Nal: Naloxone + AST 10 mg/kg; AST: Astaxanthin; Scop: Scopolamine. ^***^ *P* < 0.001 compared with control group, ^++^ *P* < 0.01, ^+++^ *P* < 0.001, compared with Scop group, and ^*P* < 0.05, ^^*P* < 0.01, ^^^*P* < 0.001 compared with astaxanthin. Data are reported as mean ± SEM.

Following the induction of the scopolamine model of AD, we observed downregulation in the expression levels of Nrf2 (*P* < 0.001, Figure 7A) and upregulation of NF-κB (*P* < 0.001, Figure 7B) proteins in the hippocampal tissue of rats. However, the administration of astaxanthin at both doses effectively reversed these alterations (*P* < 0.01). Notably, pre-treatment with the antagonists Flu and Nal mitigated the positive effects of astaxanthin (*P* < 0.05).

## 4 Discussion

**FIGURE 8:**
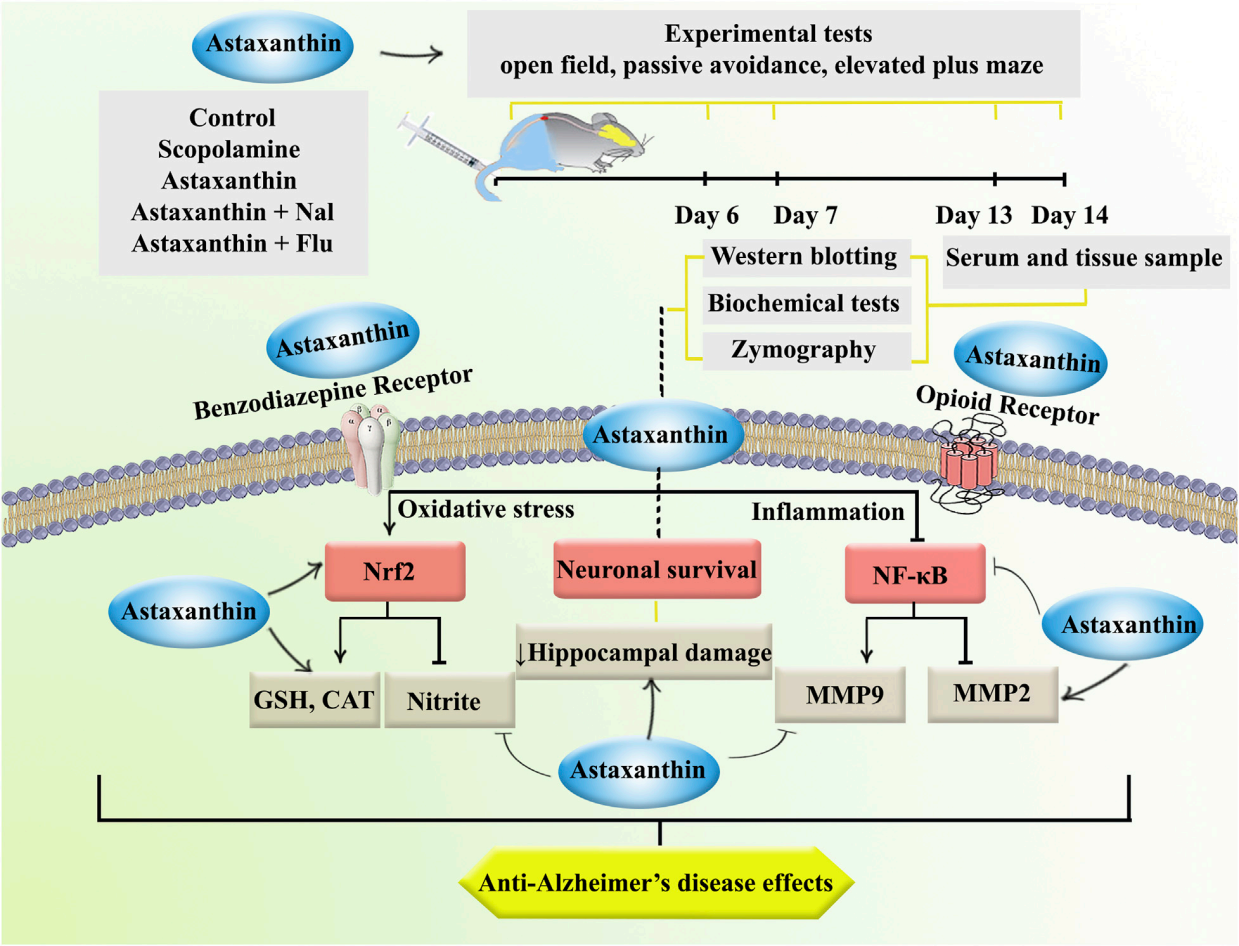
The study procedure and anti-AD effects of astaxanthin through opioid/benzodiazepine receptors by regulating Nrf2, NF-κB, and interconnected pathways.

The current study examined the effects of astaxanthin in a scopolamine-induced rat model of AD. Behavioral assessments indicated that astaxanthin alleviated AD-related cognitive deficits, including the open field, passive avoidance, and elevated plus maze tests. Furthermore, we demonstrated its antioxidant properties by restoring levels of catalase and glutathione, as well as expressing Nrf-2, which was accompanied by a reduction in the oxidative stress marker nitrite. Our findings also underscored the anti-inflammatory effects of astaxanthin, as shown by a decrease in inflammatory MMP-9, while a decrease in anti-inflammatory MMP-2 and suppression of NF-κB. These biochemical changes were further validated by histological evaluations, which highlighted the neuroprotective effects of astaxanthin. However, it is important to note that the protective effects of astaxanthin were attenuated by Nal and Flu (Figure 8).

AD remains a formidable challenge in neurology, characterized by a complex interplay of pathogenic mechanisms, including oxidative stress, neuroinflammation, and synaptic dysfunction (Kobayashi et al., 2009; McMahon et al., 2010; de Castro et al., 2019; Teixeira et al., 2019; Fakhri et al., 2020). Scopolamine acts as a non-selective muscarinic acetylcholine receptor antagonist, reducing cholinergic activity. This mimics the cholinergic deficits seen in AD patients, where impaired neurotransmission contributes to memory loss and cognitive decline. The administration of scopolamine results in several pathological changes that are characteristic of AD, including the deposition of Aβ, hyperphosphorylation of tau proteins, and increased oxidative stress levels (Chen and Yeong, 2020; Yadang et al., 2020).

In AD, elevated oxidative stress is implicated in neuronal damage, synaptic dysfunction, and the accumulation of Aβ plaques and hyperphosphorylated tau protein, contributing to the disease’s characteristic neurodegenerative processes (Zhao and Zhao, 2013; Huang et al., 2016). Nrf-2 is central to the cellular response to oxidative stress, a transcription factor that orchestrates the expression of various antioxidant and cytoprotective genes. Under normal physiological conditions, Nrf-2 is kept in the cytoplasm in an inactive form; however, under oxidative stress, it translocates to the nucleus, where it binds to antioxidant response elements (AREs) in the promoters of target genes. This process changed the expression of critical antioxidant enzymes such as catalase and glutathione (Ngo and Duennwald, 2022; Chu et al., 2024). In the brains of AD patients, the activity of antioxidant enzymes such as glutathione peroxidase is often reduced in affected brain regions, highlighting the compromised ability to counteract oxidative stress (Jurcău et al., 2022). Oxidative stress can initiate inflammatory responses by activating NF-κB. Under oxidative stress, ROS can induce the phosphorylation and subsequent degradation of IκB proteins, which normally inhibit NF-κB by sequestering it in the cytoplasm. Once IκB is degraded, NF-κB translocates to the nucleus, where it activates the transcription of pro-inflammatory genes (Lingappan, 2018). On the other hand, NF-κB can enhance the transcription of genes encoding MMPs, particularly MMP-9 (Hsieh et al., 2010). MMP-9 levels are often elevated in the brains of AD patients. It has been shown to correlate with cognitive decline and may contribute to neuroinflammation and synaptic dysfunction (Wang et al., 2014). While MMP-2 activity is decreased in AD patients and those with mild cognitive impairment, suggesting a potential protective role against Aβ accumulation (Lim et al., 2011). Those results were in line with our current study and our previous reports on the anti-inflammatory roles of MMP-2 and the inflammatory potentials of MMP-9 in AD (Zarneshan et al., 2025).

Given the critical role of oxidative stress in AD, various treatment strategies have been explored, including the use of antioxidants. Antioxidants are compounds that can mitigate oxidative damage, potentially slowing the progression of AD and improving cognitive function (Pritam et al., 2022). Phytochemicals and herbal medicines are potent antioxidants, metal chelators, and free radical scavengers, by suppressing lipid peroxidation (Gogebakan et al., 2012). For instance, there are great antioxidant properties behind the neuroprotective role of polyphenols (Daglia et al., 2014). Additionally, these compounds regulate inflammatory biomarkers, oxidative stress, and endothelial function towards neuroprotection. Of other plant secondary metabolites, iridoids showed anti-AD potentials with antioxidant effects (Hamedi et al., 2020; Kavyani et al., 2024). Natural products have also shown antioxidant properties through total antioxidant status, total oxidant status and levels, and oxidative stress index (Sevindik et al., 2020). These compounds also showed antioxidant potential by combating nitric oxide and downstream mediators (Selamoglu-Talas et al., 2015). Nutritional deficiencies are also in a near linkage with psychiatric disorders such as schizophrenia, anxiety, and depression (Vasile et al., 2024). Astaxanthin is considered one of the most powerful super antioxidants in nature, significantly more effective than vitamin C, vitamin E, and other carotenoids. Its unique molecular structure allows it to quench free radicals, reduce oxidative stress, and protect cells from damage (Fakhri et al., 2018). We previously showed the neuroprotective effects of astaxanthin (at the same doses of 5, and 10 mg/kg, i.p.) in a rat model of chronic constriction injury (Hashemi et al., 2024). Similarly, our research team confirmed the antinociceptive role of astaxanthin through nitric oxide pathway at i.p. doses of 5, and 10 mg/kg (Mohammadi et al., 2021). We also demonstrated the antioxidant properties of astaxanthin solid lipid nanoparticles (5, and 10 mg/kg, i.p.) against acute kidney injury in rats (Yarmohammadi et al., 2025). The administration of astaxanthin leads to a notable enhancement in memory and cognitive performance. A rat model of AD using hydrated aluminum chloride indicated that astaxanthin treatment can lead to a dose-dependent (5, 10, and 15 mg/kg) reduction in Aβ1-42 levels in brain tissues, thereby potentially decreasing amyloid plaque formation (Hafez et al., 2021). Magadmi et al. reported that oral administration of astaxanthin was associated with a beneficial effect on short-term memory and reduced acetylcholinesterase activity in the brain (Magadmi et al., 2024). A study utilized a vascular dementia mouse model established through unilateral common carotid artery occlusion. Mice were administered astaxanthin for 30 days, and cognitive function was assessed using the Morris water maze and object recognition tests. Results indicated that astaxanthin treatment significantly improved cognitive performance, as evidenced by increased superoxide dismutase (SOD) activity and reduced malondialdehyde (MDA) and IL-1β levels, markers of oxidative stress and inflammatory response (Zhu et al., 2020). Also, a study involving healthy adults reported that astaxanthin supplementation resulted in significant improvements in composite and verbal memory after 12 weeks compared to a placebo group. Participants noted fewer memory-related issues, such as trouble recalling names (Sekikawa et al., 2020). Astaxanthin’s unique chemical structure allows it to effectively cross the BBB, reaching CNS regions such as the hippocampus and cortex. This property is crucial for targeting neurodegenerative diseases like AD (Si and Zhu, 2022; Medoro et al., 2023). Studies using nano-emulsion formulations have quantified astaxanthin in different brain regions of animal models. For example, higher concentrations of astaxanthin were observed in the hippocampus after oral administration (160 mg/kg/day), suggesting its potential for enhancing memory-related functions (Chik et al., 2022).

Astaxanthin’s bioavailability can be influenced by its formulation, as it is a lipophilic compound. Studies have shown that astaxanthin is better absorbed when consumed with fats. Micro-encapsulation and emulsification techniques can enhance its bioavailability. A clinical trial indicated that astaxanthin can be absorbed effectively in humans, with peak plasma concentrations occurring within 1–4 h after ingestion (Mercke Odeberg et al., 2003). The elimination half-life of astaxanthin varies but is generally around 16 h in humans. This suggests that astaxanthin may require multiple dosing for sustained effects, particularly for chronic conditions like AD (Singh et al., 2020).

Our investigation revealed a significant reduction in the levels of catalase, glutathione, and MMP-2, as well as in the expression of the protein Nrf-2, in the scopolamine-treated group. Conversely, there was a notable increase in nitrite, MMP-9 levels, and the expression of the protein NF-κB.

This finding aligns with existing research indicating that scopolamine treatment can lead to oxidative stress and neuroinflammation. For instance, a study found that scopolamine downregulated antioxidant enzymes such as superoxide dismutase and catalase, while also decreasing Nrf2 expression, which is crucial for regulating antioxidant responses (Venkatesan et al., 2016). Scopolamine disrupts cholinergic signaling by antagonizing acetylcholine receptors, which can lead to reduced activation of the Nrf2 signaling pathway. Studies also suggested that scopolamine-induced cognitive impairments may be linked to its suppression of Nrf2 activity and related treatments activating Nrf2 signaling pathways (Venkatesan et al., 2016; Li et al., 2024). Scopolamine has also been linked to neuroinflammatory processes, which can further affect Nrf2 activity. Inflammatory cytokines such as tumor necrosis factor-alpha (TNF-α) and IL-6 modulate Nrf2 signaling, and scopolamine-induced inflammation may exacerbate reductions in Nrf2 expression. Some other therapeutic candidates demonstrated neuroprotective effects by reducing inflammatory markers (e.g., NF-κB and TNF-α) while increasing Nrf2 activity in a scopolamine-induced AD model (Faldu and Shah, 2024). Also, several studies have shown that scopolamine administration markedly increases oxidative stress by reducing catalase and glutathione levels, while elevating nitric oxide levels, leading to brain damage (Chen et al., 2014; Chen and Yeong, 2020; Yadang et al., 2020). Moosavi et al. found that treatment with scopolamine led to a change in the levels of MMP-2 and MMP-9 in the hippocampus (Moosavi et al., 2018). Furthermore, an increase in NF-κB expression was observed in the scopolamine-treated group (El-Marasy et al., 2018; Joseph et al., 2020).

The evidence supports that enhancing antioxidant defenses through dietary means or supplementation could play a significant role in preventing AD and managing cognitive decline (Arslan et al., 2020; Sidiropoulou et al., 2023). In further studies, we found that treatment with astaxanthin (10 mg/kg) notably restored levels of catalase, glutathione, and MMP-2, while also enhancing the expression of the protein Nrf-2. Simultaneously, it reduced nitrite levels, MMP-9 levels, and the expression of the protein NF-κB, further confirming the strong antioxidant properties of astaxanthin. Recent evidence highlighted astaxanthin’s protective role against diseases linked to oxidative stress and inflammation, focusing on its regulation of key redox-sensitive transcription factors, Nrf2 and NF-κB (Davinelli et al., 2022; Kanwugu and Glukhareva, 2023).

Opioid receptors are G-protein-coupled receptors that influence neurotransmitter release and neuronal excitability. Their signaling pathways have been implicated in the modulation of several neurodegenerative processes, including those associated with AD. Studies indicate that dysregulations in opioid receptor signaling can lead to increased expression of β-site amyloid precursor protein cleaving enzyme 1 (BACE1) and γ-secretase, both of which are crucial for the amyloidogenic pathway that produces toxic Aβ peptide (Tanguturi and Streicher, 2023). We have previously shown that Nal may diminish the potential effects of astaxanthin in pain relief (Fayazzadeh et al., 2024). A multicenter trial involving 54 subjects with clinically diagnosed AD tested the effects of intravenous Nal at doses of 1 mg, 10 mg, and even 30 mg. The results indicated no significant improvement in neuropsychological performance across these doses (Fayazzadeh et al., 2024). Another study administered Nal hydrochloride in varying doses (5 μg/kg, 0.1 mg/kg, and 2.0 mg/kg) to 12 patients with dementia of the AD type. The findings revealed no improvements in motor skills, attention, memory, or learning. Notably, some patients exhibited increased inappropriate verbal behavior and irritability after receiving the lower dose (Tariot, 1986).

GABA is crucial for regulating neuronal excitability and maintaining the balance between excitation and inhibition in the brain. In the context of AD, studies have shown that GABAergic dysfunction can lead to hyperactivity within neural networks, particularly in the hippocampus, a region critical for memory formation. This hyperactivity is often observed even before clinical symptoms manifest, suggesting that GABAergic dysregulation could serve as an early biomarker for cognitive decline associated with AD (Jiménez-Balado and Eich, 2021). Systematic reviews have consistently reported lower levels of GABA in both the brain and cerebrospinal fluid (CSF) of AD patients compared to healthy controls. For instance, a meta-analysis found standardized mean differences indicating significant reductions in GABA levels and related components such as GAD65/67 and GABA A receptors (Carello-Collar et al., 2023). Flu has been evaluated for its cognitive effects on patients with AD. Some studies suggest that while Flu might improve memory retrieval in certain contexts, it can also lead to cognitive slowing or adverse effects, indicating a complex relationship between Flu administration and cognitive performance in AD patients (Ott et al., 1996; Raut et al., 2016). Studies investigating the combination of Flu and astaxanthin suggested that pre-treatment with Flu partially blocks the anxiety-reducing effects of astaxanthin (Jayasheela et al., 2021). To investigate the underlying mechanisms contributing to the neuroprotective effects of astaxanthin, we pretreated the rats with Flu and Nal, which inhibit GABAergic and opioid pathways, respectively, before administering an effective dose of astaxanthin (10 mg/kg). The findings revealed that the neuroprotective effects of astaxanthin were significantly reduced by both Nal and Flu.

Previous research has shown that the administration of scopolamine can reduce the number of neurons in the hippocampus and disrupt normal neurogenesis, which is critical for cognition, memory, and learning (Yan et al., 2014; Lee et al., 2017). Our histological findings showed a visual reduction in hippocampal neurons, and treatment with astaxanthin partially preserved them. In experimental models, astaxanthin treatment has been linked to improved cognitive performance and a reduction in neuronal loss in mouse models of AD, attributed to the mitigation of oxidative stress and inflammation in the hippocampus (Magadmi et al., 2024).

## 5 Conclusion

The present study highlights the neuroprotective effects of astaxanthin in a scopolamine-induced rat model of AD. The results indicate that astaxanthin treatment significantly ameliorates cognitive deficits. The observed enhancements in cognitive function were accompanied by notable biochemical changes, including increased levels of antioxidant markers such as catalase and glutathione, and a reduction in nitrite levels, indicating a reduction in oxidative stress. Furthermore, astaxanthin appears to exert neuroprotective effects by modulating the expression of MMP-2, MMP-9, and the transcription factors Nrf2 and NF-κB, and preservation of neurons. Moreover, the interaction of astaxanthin with key neurotransmitter systems, specifically through the modulation of GABA-A and opioid receptors, emphasizes its multi-targeted approach in addressing the complex pathophysiology of AD. The partial reversal of astaxanthin’s effects by the antagonists Flu and Nal suggests that these pathways may significantly contribute to the neuroprotective mechanisms of astaxanthin.

While the data are promising, further research is needed to fully elucidate the precise mechanisms by which astaxanthin exerts its effects in the context of AD. Future studies should aim to investigate the long-term effects of astaxanthin administration and other mechanisms, as well as sex differences involved in the pathogenesis of AD. Additionally, providing novel delivery systems is appreciated to drawback possible pharmacokinetic limitations (Fakhri et al., 2022a).

## Data Availability

The raw data supporting the conclusions of this article will be made available by the authors, without undue reservation.
